# Heparin activation of protein Z-dependent protease inhibitor (ZPI) allosterically blocks protein Z activation through an extended heparin-binding site

**DOI:** 10.1016/j.jbc.2022.102022

**Published:** 2022-05-10

**Authors:** Xin Huang, Richard Swanson, Steven T. Olson

**Affiliations:** 1 Department of Pharmacology and Regenerative Medicine, Chicago, Illinois, USA; 2 Department of Periodontics, University of Illinois Chicago, Chicago, Illinois, USA

**Keywords:** protein Z-dependent protease inhibitor, protein Z, heparin, serpin, factor Xa, anticoagulant, AT, antithrombin, BSA, bovine serum albumin, FXa, activated factor X, HCII, heparin cofactor II, H5, pentasaccharide heparin, H50, 50-saccharide heparin, H72, 72-saccharide heparin, NBD, *N*,*N*′-dimethyl-*N*-(acetyl)-*N*’-(7-nitrobenz-3-oxa-1,3-diazol-4-yl) ethylenediamine, PCI, protein C inhibitor, PDB, Protein Data Bank, PN-1, protease nexin 1, PZ, protein Z, SI, stoichiometry of inhibition, ZPI, protein Z-dependent protease inhibitor

## Abstract

Protein Z (PZ)-dependent protease inhibitor (ZPI) is a plasma anticoagulant protein of the serpin superfamily, which is activated by its cofactor, PZ, to rapidly inhibit activated factor X (FXa) on a procoagulant membrane surface. ZPI is also activated by heparin to inhibit free FXa at a physiologically significant rate. Here, we show that heparin binding to ZPI antagonizes PZ binding to and activation of ZPI. Virtual docking of heparin to ZPI showed that a heparin-binding site near helix H close to the PZ-binding site as well as a previously mapped site in helix C was both favored. Alanine scanning mutagenesis of the helix H and helix C sites demonstrated that both sites were critical for heparin activation. The binding of heparin chains 72 to 5-saccharides in length to ZPI was similarly effective in antagonizing PZ binding and in inducing tryptophan fluorescence changes in ZPI. Heparin binding to variant ZPIs with either the helix C sites or the helix H sites mutated showed that heparin interaction with the higher affinity helix C site most distant from the PZ-binding site was sufficient to induce these tryptophan fluorescence changes. Together, these findings suggest that heparin binding to a site on ZPI extending from helix C to helix H promotes ZPI inhibition of FXa and allosterically antagonizes PZ binding to ZPI through long-range conformational changes. Heparin antagonism of PZ binding to ZPI may serve to spare limiting PZ and allow PZ and heparin cofactors to target FXa at different sites of action.

Heparin-binding protein protease inhibitors of the serpin family play an important role in regulating the activity of proteases of the blood clotting cascade. As a cofactor, heparin accelerates the inhibition of clotting proteases by the serpins, antithrombin (AT, serpin C1), heparin cofactor II (HCII, serpin D1), protease nexin 1 (PN-1, serpin E2), and protein C inhibitor (PCI, serpin A5) up to several 1000-fold (1). Because of this potent stimulating effect, heparin has long been used as an antithrombotic medication in clinical practice. The heparin-binding site of these serpins has been well characterized. Basic residues on Helix D of AT, HCII, and PN-1 mediates heparin interactions with these serpins (2, 3, 4), whereas for PCI, the heparin-binding site is mainly composed of basic residues around helix H (5, 6).

Protein Z (PZ)-dependent protease inhibitor (ZPI) (serpin A10) is a serpin that circulates in plasma as a tight complex with its cofactor, PZ, at a concentration of ∼50 nM (7, 8). ZPI plays an important anticoagulant role by rapidly inhibiting activated factor X (FXa) in the presence of PZ, procoagulant lipids, and calcium. ZPI also directly inhibits activated factor XI (9). Heparin is also a physiologically relevant cofactor for ZPI regulation of its protease targets, enhancing both ZPI–FXa and ZPI–activated factor XI reaction rates to the physiologically relevant range (10^6^–10^7^ M^−1^ s^−1^) (10).

The heparin-binding site of ZPI has been previously mapped to basic residues of helices C and D (11), a site far removed from the PZ-binding site centered on helix G (12, 13). We were therefore surprised to find in a recent study that heparin appeared to antagonize the ZPI–PZ interaction by inducing the ZPI–PZ complex to dissociate (14). This suggested that heparin might actually bind to an additional site closer to the PZ-binding site so as to interfere with PZ binding. In the present study, we have used web-based computer protein–ligand docking tools together with mutagenesis to demonstrate a novel extended heparin-binding site on ZPI that extends from the previously mapped site in helix C to helix H and which is critical for heparin activation of ZPI anticoagulant function. We in addition exploit intrinsic and extrinsic fluorophore reporters on ZPI to provide evidence that heparin binding to ZPI interferes with PZ binding not by proximity effects but through allosterically induced conformational changes in ZPI, which weaken PZ binding. The implications for how heparin and PZ cofactors regulate ZPI anticoagulant function under physiological conditions are discussed.

## Results

### Heparin is an antagonist of the ZPI–PZ interaction

**Figure 1:**
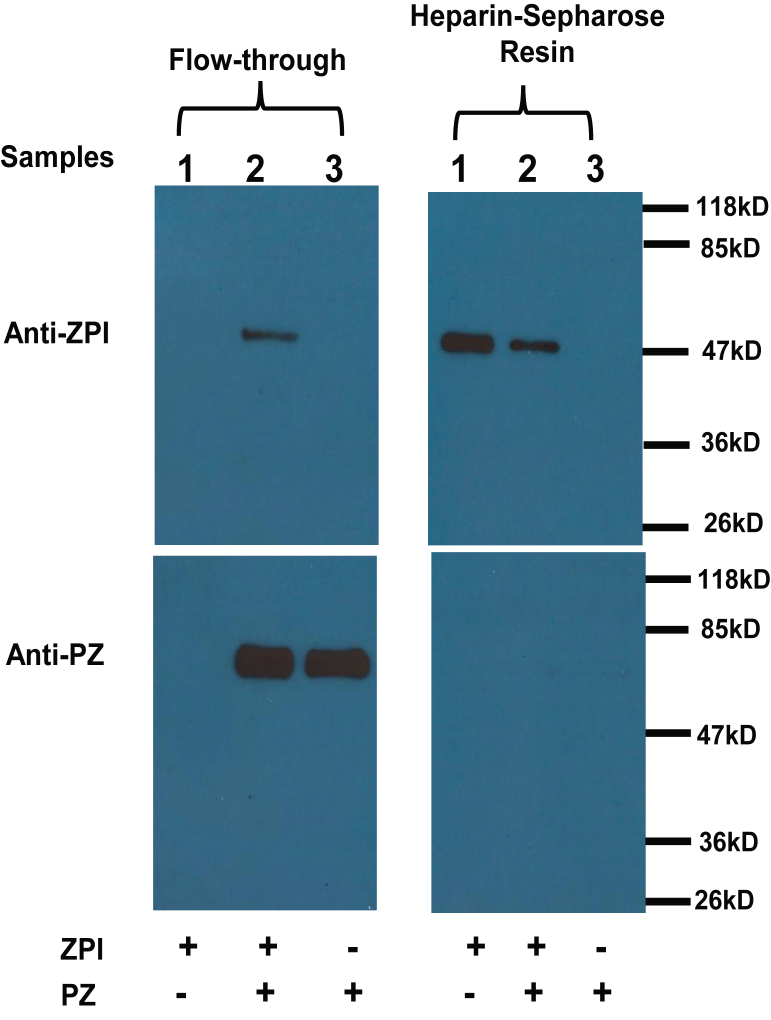
**Immunoblotting analysis of ZPI, PZ, and ZPI–PZ complex interactions with heparin-Sepharose.** About 500 nM ZPI, ZPI–PZ (500 nM each), or 500 nM PZ in 200 μl of 50 mM Tris buffer, pH 7.4, 0.1 M NaCl, and 2.5 mM CaCl_2_ buffer at 25 ^°^C were mixed with 50 μl heparin-Sepharose resin pre-equilibrated in the same buffer. The mixture was transferred to a small column with the outlet closed, incubated for 40 min, and unbound proteins then eluted followed by extensive washing of the resin with reaction buffer. The flowthrough and resin were mixed with SDS-sample buffer, boiled, and subjected to 10% SDS-PAGE followed by immunoblotting analysis with ZPI or PZ antibodies as described in the “Experimental procedures” section. PZ, protein Z; ZPI, protein Z-dependent protease inhibitor.

In a recent study, we analyzed ZPI–PZ complex formation in the absence and presence of a 50-saccharide heparin (H50) representing the average chain length of naturally occurring heparin by gel-filtration chromatography and surprisingly found that heparin had caused the ZPI–PZ complex to dissociate (14). To further substantiate the antagonistic action of heparin on the ZPI–PZ interaction, we applied the proteins alone and in complex to a small column of heparin-Sepharose resin followed by eluting the unbound proteins with physiologic buffer. As expected, applying ZPI alone resulted in the detection of ZPI on the heparin-Sepharose resin and not in the flowthrough (sample 1, Fig. 1), indicating its complete binding to the immobilized heparin. In contrast, applying PZ alone resulted in the detection of PZ only in the flowthrough and not on the resin (sample 3), indicating that PZ does not bind heparin-Sepharose. When ZPI–PZ complex was applied to the heparin-Sepharose column, only ZPI was detected on the resin, whereas PZ was found only in the flowthrough along with a trace of ZPI (sample 2), indicating that even when complexed with ZPI, PZ is unable to bind heparin-Sepharose through its high-affinity interaction with ZPI.

**Figure 2:**
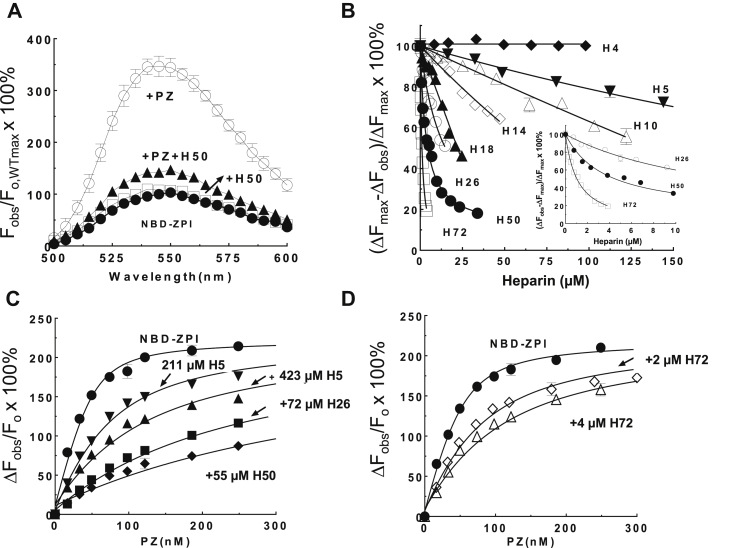
**Heparin antagonizes PZ binding to ZPI.***A*, fluorescence emission spectra of 50 nM K239C-NBD-ZPI were measured alone (**●)** after addition of saturating PZ (100 nM) (○), after further addition of 35 μM 50-saccharide (H50) heparin (**▲**), or after addition of the same concentration of H50 in the absence of PZ (□). Spectra were obtained over the emission range of 500 to 600 nm with excitation at 480 nm, in triplicate and averaged. *B*, titrations of the NBD-ZPI–PZ complex (50 nM ZPI/62 nM PZ) with heparins ranging in length from 4 to 72 saccharides (H4 ♦, H5 ▼, H10 Δ, H14 ◊, H18 ▲, H26 ○, H50 ●, and H72 □) followed by the quenching of the enhanced fluorescence of the NBD-ZPI–PZ complex measured at 480 nm excitation and 540 nm emission wavelengths. *Solid lines* show fits of the titrations by the competitive binding equation from which equilibrium dissociation constants for NBD-ZPI–heparin interactions were obtained (Table 1). *Inset graph* shows data for 26- to 72-saccharide heparins in the low heparin concentration range. *C* and *D*, titrations of 50 nM NBD-ZPI with PZ in the absence (●) or presence of 211 μM (▼) or 422 μM (▲) H5, (♦) 55 μM H50, or (■) 72 μM H26 in *C* or 2 μM (◊) or 4 μM (Δ) H72 in *D* were monitored by the ∼300% enhancement in fluorescence of NBD-ZPI. *Solid lines* are fits of titration data by the quadratic equilibrium binding equation from which apparent *K*_*D*_s for the ZPI–PZ interaction were obtained (Table 2). Data in *A*–*D* represent the average of three to five independent measurements, presented as mean ± SD. Some SD lines lie within the symbol. Further details are provided in the “Experimental procedures” section. PZ, protein Z; ZPI, protein Z-dependent protease inhibitor.

We further analyzed ZPI, PZ, and heparin interactions using a single cysteine ZPI mutant (Lys 239 Cys) labeled with the fluorophore, *N*,*N*′-dimethyl-*N*-(acetyl)-*N*’-(7-nitrobenz-3-oxa-1,3-diazol-4-yl) ethylenediamine (NBD), a probe we developed previously (15). Residue 239 is located on the periphery of the ZPI–PZ binding interface, and an NBD label at this position is highly sensitive to PZ binding as reflected by the ∼300% enhancement in NBD fluorescence when saturating PZ is added to the labeled ZPI (Fig. 2*A*). By contrast, addition of the H50 to the NBD-labeled ZPI at levels sufficient to saturate the protein caused minimal changes in NBD fluorescence, implying that NBD at position 239 does not sense heparin binding and is thus more distant from the heparin-binding site. When the equivalent saturating level of H50 was added to NBD-labeled ZPI complexed with PZ, however, a nearly complete quenching of the enhanced NBD fluorescence of the complexed ZPI was observed, suggesting that heparin binding to the NBD–ZPI/PZ complex had caused the dissociation of PZ (Fig. 2*A*). Titrations of the NBD–ZPI–PZ complex with naturally occurring 26- to 72-saccharide heparins (H72) as well as oligosaccharide heparin chains ranging from 18 to four saccharides showed that all except the 4-saccharide heparin were able to dose-dependently and saturably quench the fluorescence signal of the NBD–ZPI/PZ complex in accordance with a competitive binding model (Fig. 2*B*). Full-length and oligosaccharide heparin chains as small as a pentasaccharide were thus all effective at competitively displacing PZ from its complex with ZPI. Progressively higher heparin concentrations were necessary to achieve similar levels of quenching as the heparin chain length decreased, indicating that heparin binding to ZPI displaced PZ from the ZPI–PZ complex with an affinity dependent on heparin chain length, in keeping with past findings (10). Fitting these titrations with the competitive binding equation confirmed that *K*_*D*_s for heparin binding to NBD–ZPI increased from ∼0.1 μM for the H72 to ∼30 μM for the pentasaccharide heparin (H5) (Table 1). These values compare well with values kinetically determined from the concentration dependence of the heparin-accelerating effect on heparin concentration in our previous study (10).

**Table 1:** Chain length–dependent affinities of oligosaccharide and full-length heparins for ZPI derived from competitive binding titrations

Heparin chain length (saccharides)	ZPI–heparin
	*K*_*D*,H_ (μM)
4	Not measurable
5	32.4 ± 1.9
10	15.9 ± 1.6
14	8.1 ± 0.6
18	2.3 ± 0.3
26	1.2 ± 0.06
50	0.3 ± 0.01
72	0.07 ± 0.006

Dissociation constants (*K*_*D*,H_) for ZPI interaction with heparins of varying chain length were determined by fitting competitive binding titrations of NBD-ZPI–PZ complex with heparin in Figure 2*B* by the equation in the “Experimental procedures” section.

Competition between heparin and PZ for binding to ZPI was further demonstrated by titrating NBD-labeled ZPI with PZ in the absence and presence of fixed levels of H5 or full-length heparin chains (Fig. 2, *C* and *D*). Fitting of the titration data by the binding equation showed that the apparent PZ-binding affinity was progressively decreased as the fixed heparin concentration was increased (Table 2). *K*_*D*_s inferred for ZPI–heparin interactions from the observed decreases in PZ-binding affinity assuming a competitive binding model compared reasonably well with those determined by direct heparin-binding fluorescence titrations of unlabeled ZPI under the higher ionic strength conditions of these experiments.

**Table 2:** Heparin dose-dependently weakens the affinity of PZ for NBD-ZPI

Heparin	(Heparin)_o_ (μM)	ZPI–PZ	ZPI–heparin
		*K*_*D*,PZapp_ (nM)	*K*_*D*,H_ (μM)
None		9.0 ± 1.2	
H5	211	44.8 ± 3.7	53
H5	423	95.6 ± 12.2	44
H72	2	52.3 ± 4.9	0.42
H72	4	83.5 ± 10.1	0.48
H26	72	218.0 ± 15.0	3.1
H50	55	415.0 ± 42.0	1.2

Apparent dissociation constants for NBD-ZPI–PZ interactions (*K*_*D*,PZapp_) in the absence and presence of varying chain-length heparins were measured from fits of titrations of NBD-ZPI with PZ in Figure 2, *C* and *D*. Predicted *K*_*D*,H_s for ZPI–heparin interactions were calculated based on the relation, *K*_*D*,PZapp_= *K*_*D*,PZ_ × (1 + [H]_o_/*K*_*D*,H_), given in the “Experimental procedures” section.

### ZPI protein fluorescence is perturbed by heparin binding

**Figure 3:**
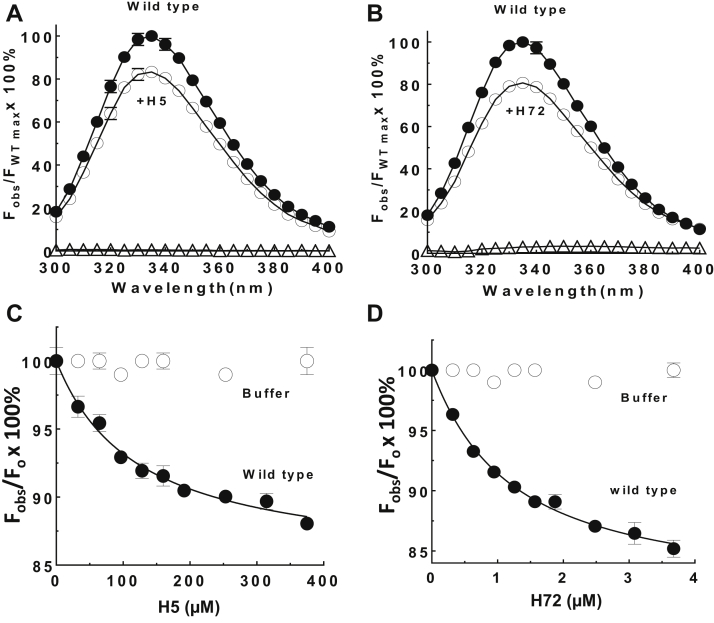
**Heparin quenches ZPI intrinsic protein fluorescence.***A* and *B*, intrinsic protein fluorescence emission spectra of 200 nM WT ZPI were measured in the absence (**●**) and presence of 400 μM H5 (*A*, ○) or 4 μM H72 (*B*, ○) over the emission range of 300 to 400 nm with excitation at 280 nm. Spectra of the same concentrations of H5 (*A*, Δ) or H72 (*B*, Δ) in the absence of ZPI were also obtained. The spectra shown are averages of triplicate spectra. *C* and *D*, equilibrium dissociation constants for WT ZPI interactions with H5 or H72 were measured by titrating H5 (*C*, **●)** or H72 (*D*, **●**) into 200 nM ZPI and monitoring the changes in protein fluorescence signal at 340 nm (excitation, 280 nm). Control titrations are shown in which the same volume of buffer as heparin was added to ZPI after correction for dilution (○). Data in *A*–*D* represent the average of three to five independent measurements, presented as mean ± SD. Some SD lines lie within the symbol. Measurements were made in 20 mM Tris, 0.2 M NaCl, 0.1% PEG 8000, and 2.5 mM CaCl_2_ buffer, pH 7.4, at 25 ^°^C. Further details are provided in the “Experimental procedures” section. ZPI, protein Z-dependent protease inhibitor.

Heparin binding to ZPI was found to be accompanied by changes in ZPI intrinsic protein fluorescence. Addition of near saturating levels of either the H5 or the full-length H72 to ZPI resulted in a similar 15 to 20% quenching of the protein fluorescence emission spectrum of the protein (Fig. 3, *A* and *B*). Titrations of ZPI with the H5s or the H72s showed that the fluorescence quenching was saturable and could be fit by the quadratic binding equation to yield a maximal quenching of −15 ± 1% and *K*_*D*_ of 123 ± 23 μM for H5 binding and quenching of −19 ± 1% and *K*_*D*_ of 1.1 ± 0.1 μM for H72 binding (Fig. 3, *C* and *D*).

### ZPI–heparin docking analysis predicts two favored heparin-binding sites

**Figure 4:**
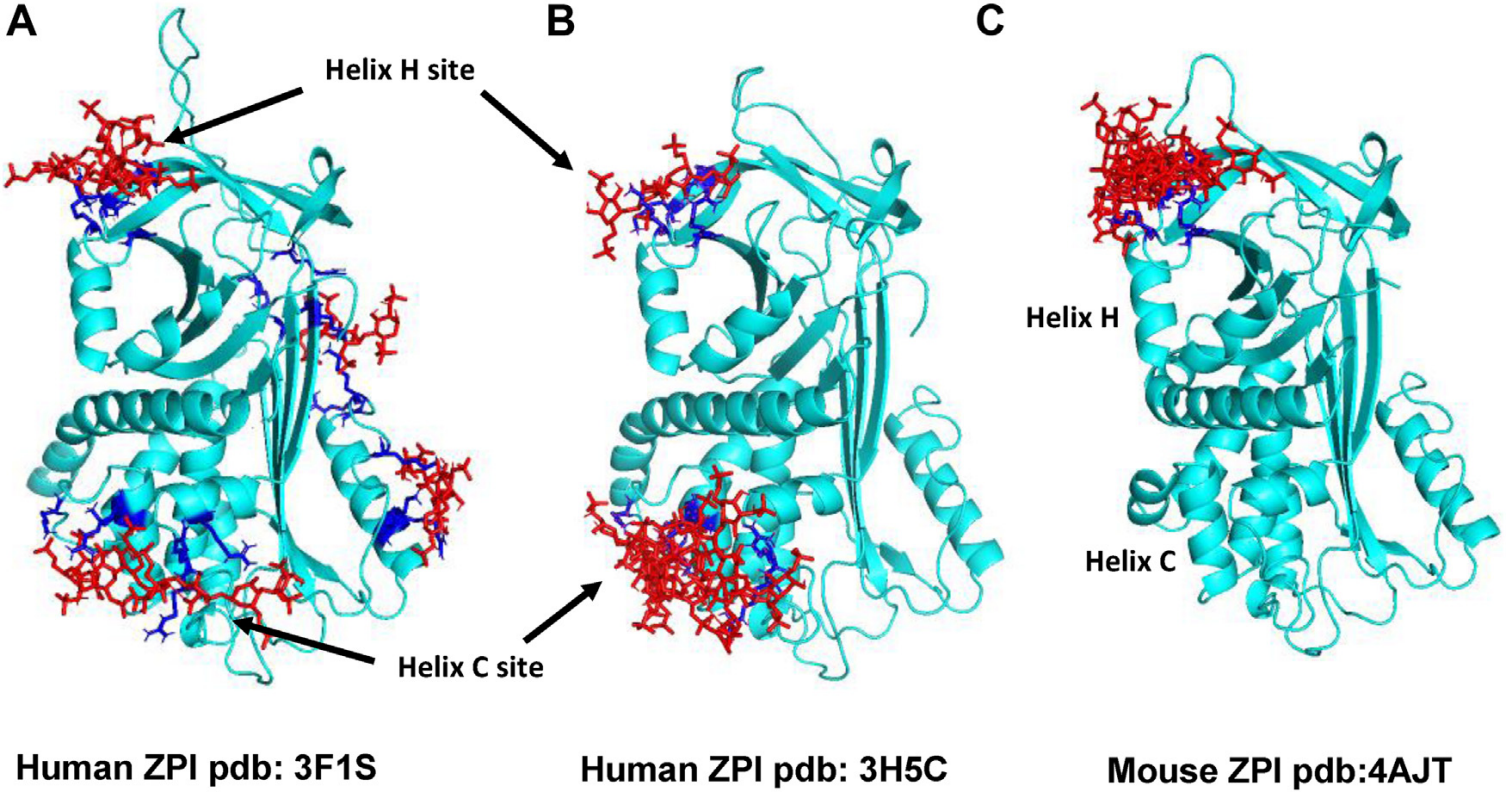
**Virtual docking of a heparin tetrasaccharide to human and mouse ZPIs.** The ZPI chains of two human ZPI–PZ complex crystal structures (colored *cyan*, *A* and *B*) and the uncomplexed mouse ZPI structure (*C*) were virtually docked to a heparin tetrasaccharide (*red stick*) with the program, CLUSPRO. One human ZPI structure (PDB code: 3F1S, *left*) predicted binding at four favored sites with a site near helix H most favored (K260, R263, K308, R310, 40% of models) followed by a helix C site (K104, R105, K113, R337, R338, 16% of models). The other human ZPI structure (PDB code: 3H5C, *left*) predicted the most favorable binding at the helix C site (K104, R105, K113, K116, R332, R337, R338, 92% of models) but also predicted the helix H site (K260, R263, K308, R310, 5% of models). The uncomplexed mouse ZPI structure predicted binding solely at the helix H site (K260, R263, K308, R310 [human numbering], >99% of models). ZPI basic residues predicted to bind heparin are in *blue stick*. PDB, Protein Data Bank; PZ, protein Z; ZPI, protein Z-dependent protease inhibitor.

**Figure 5:**
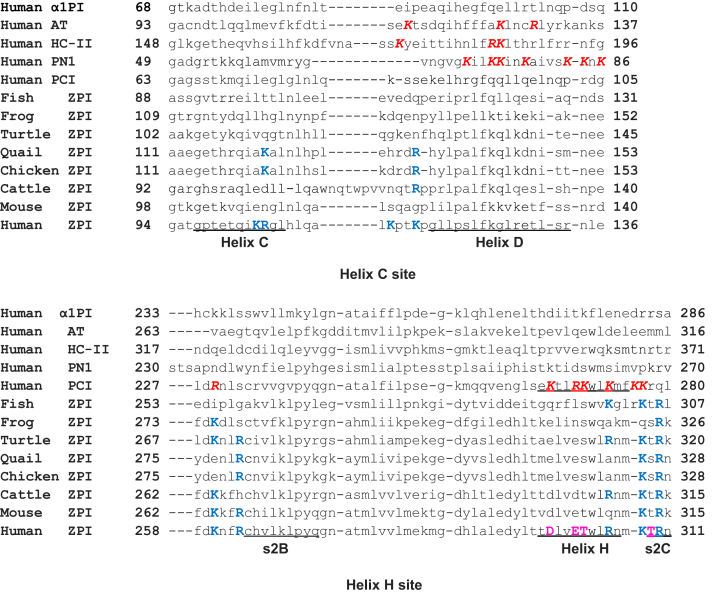
**Sequence alignments of the heparin-binding sites of heparin-activated serpins.** Shown are the alignments of the identified heparin-binding residues (in *bold italic caps colored red*) of the human serpins, antithrombin (AT): K114, K125, and R129; heparin cofactor II (HC-II): K173, R184, and K185; protease nexin-1 (PN-1): K71, K74, K75, K78, K83, K84, and K86; and protein C inhibitor (PCI): R229, K266, R269, K270, K273, K276, and K277. Below these sequences are aligned several vertebrate ZPI sequences with the identified heparin-binding residues of human ZPI colored *blue in caps*. The latter include the helix C site residues: K104, R105, K113, and K116; and the helix H site residues: K260, R263, R305, K308, and R310. ZPI homologs of PCI heparin-binding residues, which were changed back to PCI residues, are in *purple colored caps*: D298, E301, T302, and T309. ZPI, protein Z-dependent protease inhibitor.

The aforementioned findings clearly demonstrated that heparin binding to ZPI antagonizes PZ binding. This was surprising given that a previous mapping of the ZPI heparin-binding site implicated a site distant from the PZ binding site (11). The previous observations that mutation of basic residues in this site did not completely abrogate heparin binding to ZPI or heparin acceleration of ZPI inhibition of FXa, however, suggested that an additional basic site on ZPI for heparin binding might exist close to the PZ-binding site so as to interfere with PZ binding. To assess this possibility, we employed the docking program, CLUSPRO (http://cluspro.bu.edu/) (16), to predict the most likely basic regions of ZPI to bind a tetrasaccharide heparin. Inputting the native ZPI chains of the two human ZPI–PZ complex structures (Protein Data Bank [PDB] code: 3F1S (12) and 3H5C (13)) that have been reported resulted in two favored heparin-binding sites, one in the helix C site previously mapped and another in a novel site close to helix H and adjacent to the PZ-binding site (Fig. 4). Notably, the latter site is immediately adjacent to the helix H site for heparin binding to the related A clade serpin, PCI (5, 6). Surprisingly, when the docking analysis was performed with the mouse uncomplexed native ZPI structure (PDB code: 4AJT) (17), only the helix H site was predicted. Alignment of human and mouse ZPI sequences showed that the reason for this was that the helix C site is not conserved in the mouse sequence. Alignment of other ZPI vertebrate sequences confirmed a strong conservation of the helix H basic residues and lack of conservation of the helix C site (Fig. 5).

### Validation of the predicted heparin-binding site by mutagenesis

To determine whether the predicted helix H-binding site for heparin on ZPI was involved in heparin cofactor function, we proceeded to mutate the residues in the predicted site singly and in combination and analyze how the mutations affected the ability of heparin to accelerate ZPI inhibition of FXa. Because ZPI is not a completely efficient inhibitor of FXa in the absence or the presence of heparin or PZ cofactors and can be cleaved by FXa as a substrate in a competing reaction, as reflected by stoichiometries of inhibition (SIs) greater than 1, kinetic evaluations of ZPI–FXa reactions must correct the apparent rate constant for ZPI inhibition of FXa by the extent of this substrate reaction by multiplying by the SI (18).

As a control, we first determined whether the helix H site mutations affected the PZ cofactor function of ZPI. Table 3 shows that PZ produced a robust acceleration of FXa inhibition by the helix H mutant ZPIs in the presence of phospholipid and calcium relative to unaccelerated rates of inhibition, demonstrating that the mutations minimally perturbed PZ activation of ZPI anticoagulant function. By contrast, a D293A ZPI mutation in the PZ-binding site greatly abrogated the acceleration, in keeping with our previous demonstration that D293 is a hot spot residue for PZ binding (17).

**Table 3:** Second-order association rate constants and inhibition stoichiometries for unaccelerated and PZ-accelerated reactions of WT and mutant ZPIs with FXa

ZPI mutant	Unaccelerated reactions				PZ-accelerated reactions				PZ enhancement^a^
	*k*_a,app_ (M^−1^ s^−1^)	SI	*k*_a,app_ × SI (M^−1^ s^−1^)		*k*_a,app_ (M^−1^ s^−1^)	SI	*k*_a,app_ × SI (M^−1^ s^−1^)		
WT	5.6 ± 0.3 × 10^3^	2.8 **±** 0.4	1.6 **±** 0.3 × 10^4^	(1)	10.9 ± 0.1 × 10^6^	4.0 **±** 0.3	4.4 ± 0.3 × 10^7^	(1.0)	2750
D293A	4.1 ± 0.2 × 10^3^	2.6 ± 0.1	1.1 ± 0.1 × 10^4^	(0.69)	−	−	−		−
Helix H site mutations									
K253A	4.1 ± 0.4 × 10^3^	2.1 ± 0.1	8.5 ± 1.2 × 10^3^	(0.53)	3.0 ± 0.2 × 10^6^	2.4 ± 0.1	7.2 ± 0.1 x 10^6^	(0.16)	847
K260A	4.8 ± 0.3 × 10^3^	2.2 ± 0.2	1.0 ± 0.2 × 10^4^	(0.62)	8.9 ± 0.2 × 10^6^	3.1 ± 0.1	2.6 ± 0.2 x 10^7^	(0.59)	2600
R263A	2.4 ± 0.1 × 10^3^	2.7 ± 0.5	6.4 ± 1.3 × 10^3^	(0.40)	6.9 ± 0.5 × 10^6^	3.1 ± 0.1	2.1 ± 0.2 x 10^7^	(0.48)	3359
K284A	5.0 ± 0.3 × 10^3^	2.3 ± 0.2	1.2 ± 0.2 × 10^4^	(0.75)	6.2 ± 0.1 × 10^6^	3.3 ± 0.1	2.0 ± 0.1 × 10^7^	(0.45)	1733
R305A	7.3 ± 0.4 × 10^3^	2.4 ± 0.2	1.8 ± 0.2 × 10^4^	(1.1)	9.8 ± 1.0 × 10^6^	3.3 ± 0.1	3.2 ± 0.4 × 10^7^	(0.73)	1778
K308A	5.8 ± 0.1 × 10^3^	2.9 ± 0.2	1.7 ± 0.1 × 10^4^	(1.1)	9.5 ± 0.1 × 10^6^	2.7 ± 0.1	2.6 ± 0.1 × 10^7^	(0.59)	1524
R310A	3.8 ± 0.3 × 10^3^	2.7 ± 0.2	1.0 ± 0.2 × 10^4^	(0.62)	9.4 ± 1.4 × 10^6^	2.9 ± 0.2	2.7 ± 0.6 × 10^7^	(0.61)	2700
K260A/R263A	5.4 ± 0.3 × 10^3^	2.4 ± 0.5	1.3 ± 0.4 × 10^4^	(0.81)	5.6 ± 0.2 × 10^6^	2.6 ± 0.3	1.5 ± 0.2 × 10^7^	(0.34)	1153
K284A/R310A	3.3 ± 0.3 × 10^3^	1.9 ± 0.2	6.3 ± 1.3 × 10^3^	(0.39)	6.4 ± 0.4 × 10^6^	3.3 ± 0.1	2.1 ± 0.2 × 10^7^	(0.48)	3317
K260A/R263A/K308A/R310A	1.1 ± 0.2 × 10^3^	4.0 ± 0.8	4.5 ± 1.8 × 10^3^	(0.28)	4.1 ± 0.3 × 10^6^	6.6 ± 0.4	2.7 ± 0.4 × 10^7^	(0.61)	6000
K260A/R263A/R305A/K308A/R310A	2.6 ± 0.1 × 10^3^	2.9 ± 0.3	7.5 ± 0.9 × 10^3^	(0.47)	3.9 ± 0.1 × 10^6^	4.5 ± 0.2	1.8 ± 0.1 × 10^7^	(0.41)	2400
K253A/K260A/R263A/R305A/K308A/R310A	9.2 ± 0.6 × 10^2^	3.2 ± 0.1	2.9 ± 0.2 × 10^3^	(0.18)	3.4 ± 0.1 × 10^6^	7.4 ± 0.3	2.5 ± 0.2 × 10^7^	(0.57)	8620
Helix C site mutations									
K125A/R128A	3.7 ± 0.1 × 10^3^	1.7 ± 0.1	6.3 ± 0.3 × 10^3^	(0.39)	5.2 ± 0.2 × 10^6^	5.6 ± 0.1	2.9 ± 0.2 × 10^7^	(0.66)	4603
K104A/R105A/K113A/K116A	5.9 ± 0.1 × 10^3^	2.4 ± 0.2	1.4 ± 0.2 × 10^4^	(0.88)	10.1 ± 1.9 × 10^6^	2.3 ± 0.1	2.3 ± 0.5 × 10^7^	(0.52)	1642
K104A/R105A/K113A/K116A/K125A/R128A	2.8 ± 0.1 × 10^3^	3.4 ± 0.4	9.4 ± 1.6 × 10^3^	(0.59)	1.1 ± 0.1 × 10^6^	7.7 ± 0.1	8.5 ± 0.9 × 10^6^	(0.19)	904
PCI helix H mutant									
D298K/E301R/T302K/T309K	7.3 ± 0.1 × 10^3^	3.6 ± 0.3	2.6 ± 0.2 × 10^4^	(1.6)	8.4 ± 1.2 × 10^6^	2.2 ± 0.1	1.8 ± 0.3 × 10^7^	(0.41)	692

Apparent second-order association rate constants (*k*_a,app_) and SI (moles ZPI/mole FXa) for ZPI–FXa reactions were measured in the absence of PZ or in the presence of equimolar PZ plus 25 μM phospholipid, 5 mM CaCl_2_ in 20 mM Tris, 0.1 M NaCl buffer (pH 7.4) at 25 °C as described under the “Experimental procedures” section.

Buffer contained 2.5 mM CaCl_2_ for reactions in the absence of PZ. The product of *k*_a,app_ and SI represents the corrected association rate constant for reaction through the inhibitory pathway with values in parentheses expressing these rate constants relative to WT.

a Ratio of PZ-accelerated to unaccelerated values of *k*_a,app_ × SI.

**Figure 6:**
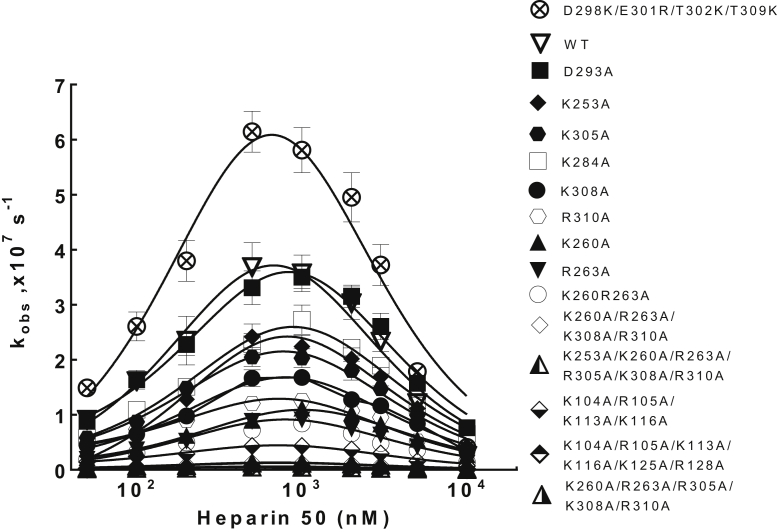
**Dependence of 50-saccharide heparin (H50) accelerating effects on ZPI inactivation of FXa on H50 concentration.** The pseudo–first-order rate constant (*k*_obs_) for reaction of 70 nM WT or mutant ZPIs with 0.5 nM FXa is plotted as a function of H50 concentration. Reactions were performed in 20 mM Tris buffer (pH 7.4), 0.1 M NaCl, 0.1 mg/ml BSA, 0.1% PEG 8000, and 2.5 mM CaCl_2_ at 25 ^°^C. *k*_obs_ was calculated from the fractional loss of FXa activity at different reaction times and the mean of triplicate measurements ± SD plotted as described in the “Experimental procedures” section. *Solid lines* are computer fits of data by the template bridging model. BSA, bovine serum albumin; FXa, activated factor X; ZPI, protein Z-dependent protease inhibitor.

Full-length heparins accelerate the ZPI–FXa reaction through a template bridging mechanism, which is reflected by the bell-shaped dependence of the acceleration on heparin concentration. The maximum acceleration of the WT ZPI reaction by H50 occurs at about 1 μM heparin under physiologic conditions (Fig. 6). Analysis of the kinetics of H50 acceleration of mutant ZPI–FXa reactions as a function of heparin concentration showed a similar bell-shaped dependence and optimal acceleration at ∼1 μM heparin. Notably, several single and combined mutations in the predicted helix H site significantly reduced the extent of heparin acceleration of the ZPI–FXa reaction, suggesting that the predicted site was indeed important for heparin cofactor function (Fig. 6).

We proceeded to compare the effects of the ZPI mutations on H50-accelerated and unaccelerated second-order inhibition rate constants for ZPI–FXa reactions at the optimal heparin concentration of 1 μM. Table 4 tabulates the measured apparent rate constants (*k*_a,app_), SIs, and corrected inhibition rate constants for WT and all mutant ZPI reactions with FXa in the presence of the H50. H50 accelerated WT ZPI inhibition of FXa ∼75-fold over the unaccelerated rate of inhibition (Tables 3 and 4). The accelerating effect was unaffected for the K284A mutation (close to D293) and modestly reduced to 56-fold for the K253A mutant and to 55-fold for the R263A mutant. However, more significant reductions in heparin acceleration from 75-fold to ∼27-fold for K260A, ∼29-fold for R305A, ∼32-fold for K308A, and ∼35-fold for R310A were observed. Notably, mutation of the hot spot PZ-binding residue, D293A, produced a WT-like heparin acceleration, indicating that loss of PZ-binding function did not affect heparin cofactor function. While combining the K284A and R310A mutations did not further reduce the acceleration from that of the single R310A mutation (∼59-fold), the double mutation, K260A/R263A, strongly reduced the acceleration from 75-fold to ∼22-fold, implying a cumulative effect of these latter two residues. Combining the K260A/R263A double mutation with K308A and R310A mutations produced a somewhat further cumulative reduction in the quadruple mutant K260A/R263A/K308A/R310A acceleration to ∼17-fold. This reduction was further augmented to a 6.5-fold heparin acceleration by adding the R305A mutation to yield the quintuple mutant, K260A/R263A/R305A/K308A/R310A. The addition of the nearby K253A mutation to the quintuple mutant to yield the sextuple mutant, K253A/K260A/R263A/R305A/K308A/R310A, produced no further reduction (approximately ninefold) in the heparin acceleration, suggesting that the five residues in the quintuple mutant were sufficient to produce the maximum reduction in heparin acceleration of >90%. The five residues, K260, R263, R305, K308, and R310, thus appear to constitute a cluster critical for heparin activation of ZPI to inhibit FXa, in agreement with the heparin-binding site predicted by CLUSPRO (Fig. 4).

**Table 4:** Second-order association rate constants and inhibition stoichiometries for heparin-accelerated reactions of ZPI with FXa

ZPI mutant	*k*_a,app_ (M^−1^ s^−1)^	SI	*k*_a,app_ × SI (M^−1^ s^−1)^		H50 rate enhancement^a^
WT	5.1 ± 0.4 × 10^5^	2.4 **±** 0.1	1.2 **±** 0.2 × 10^6^	(1)	75
D293A	5.0 ± 0.2 × 10^5^	1.7 ± 0.1	8.5 ± 0.6 × 10^5^	(0.71)	77
Helix H site mutations					
K253A	3.2 ± 0.2 × 10^5^	1.5 ± 0.1	4.8 ± 0.7 × 10^5^	(0.40)	56
K260A	1.6 ± 0.2 × 10^5^	1.7 ± 0.2	2.7 ± 0.6 × 10^5^	(0.22)	27
R263A	1.3 ± 0.1 × 10^5^	2.8 ± 0.1	3.5 ± 0.4 × 10^5^	(0.29)	55
K284A	3.9 ± 0.2 × 10^5^	2.4 ± 0.1	9.2 ± 0.9 × 10^5^	(0.77)	77
R305A	2.9 ± 0.3 × 10^5^	1.8 ± 0.1	5.3 ± 0.9 × 10^5^	(0.44)	29
K308A	2.4 ± 0.1 × 10^5^	2.2 ± 0.3	5.4 ± 0.9 × 10^5^	(0.45)	32
R310A	1.8 ± 0.1 × 10^5^	1.9 ± 0.2	3.5 ± 0.5 × 10^5^	(0.29)	35
K260A/R263A	1.2 ± 0.1 × 10^5^	2.5 ± 0.4	2.9 ± 0.8 × 10^5^	(0.24)	22
K284A/R310A	2.0 ± 0.1 × 10^5^	1.9 ± 0.1	3.7 ± 0.4 × 10^5^	(0.31)	59
K260A/R263A/K308A/R310A	1.8 ± 0.1 × 10^4^	4.1 ± 0.6	7.6 ± 1.3 × 10^4^	(0.06)	17
K260A/R263A/R305A/K308A/R310A	1.7 ± 0.1 × 10^4^	2.9 ± 0.4	4.9 ± 0.7 × 10^4^	(0.04)	6.5
K253A/K260A/R263A/R305A/K308A/R310A	6.4 ± 0.5 × 10^3^	4.0 ± 0.2	2.6 ± 0.3 × 10^4^	(0.02)	9.0
Helix C site mutations					
K125A/R128A	1.2 ± 0.1 × 10^5^	4.9 ± 0.2	5.6 ± 0.8 × 10^5^	(0.47)	89
K104A/R105A/K113A/K116A	6.2 ± 0.1 × 10^4^	1.9 ± 0.2	1.2 ± 0.1 × 10^5^	(0.10)	8.6
K104A/R105A/K113A/K116A/K125A/R128A	8.5 ± 1.3 × 10^3^	7.8 ± 2.0	6.6 ± 2.7 × 10^4^	(0.06)	7.0
PCI helix H mutant					
D298K/E301R/T302K/T309K	8.3 ± 0.4 × 10^5^	4.6 ± 0.1	3.9 ± 0.2 × 10^6^	(3.2)	150

Apparent second-order association rate constants (*k*_a,app_) and SI (moles ZPI/mole FXa) for H50-accelerated reactions of ZPI with FXa were measured at the optimal heparin concentration of 1 μM in 20 mM Tris, 0.1 M NaCl, 2.5 mM CaCl_2_ buffer (pH 7.4) at 25 °C as described under the “Experimental procedures” section.

The product of *k*_a,app_ and SI represents the corrected association rate constant for reaction through the inhibitory pathway with values in parentheses expressing these rate constants relative to WT.

a Ratio of heparin-accelerated to unaccelerated values of *k*_a,app_ × SI (the latter from Table 3).

**Figure 7:**
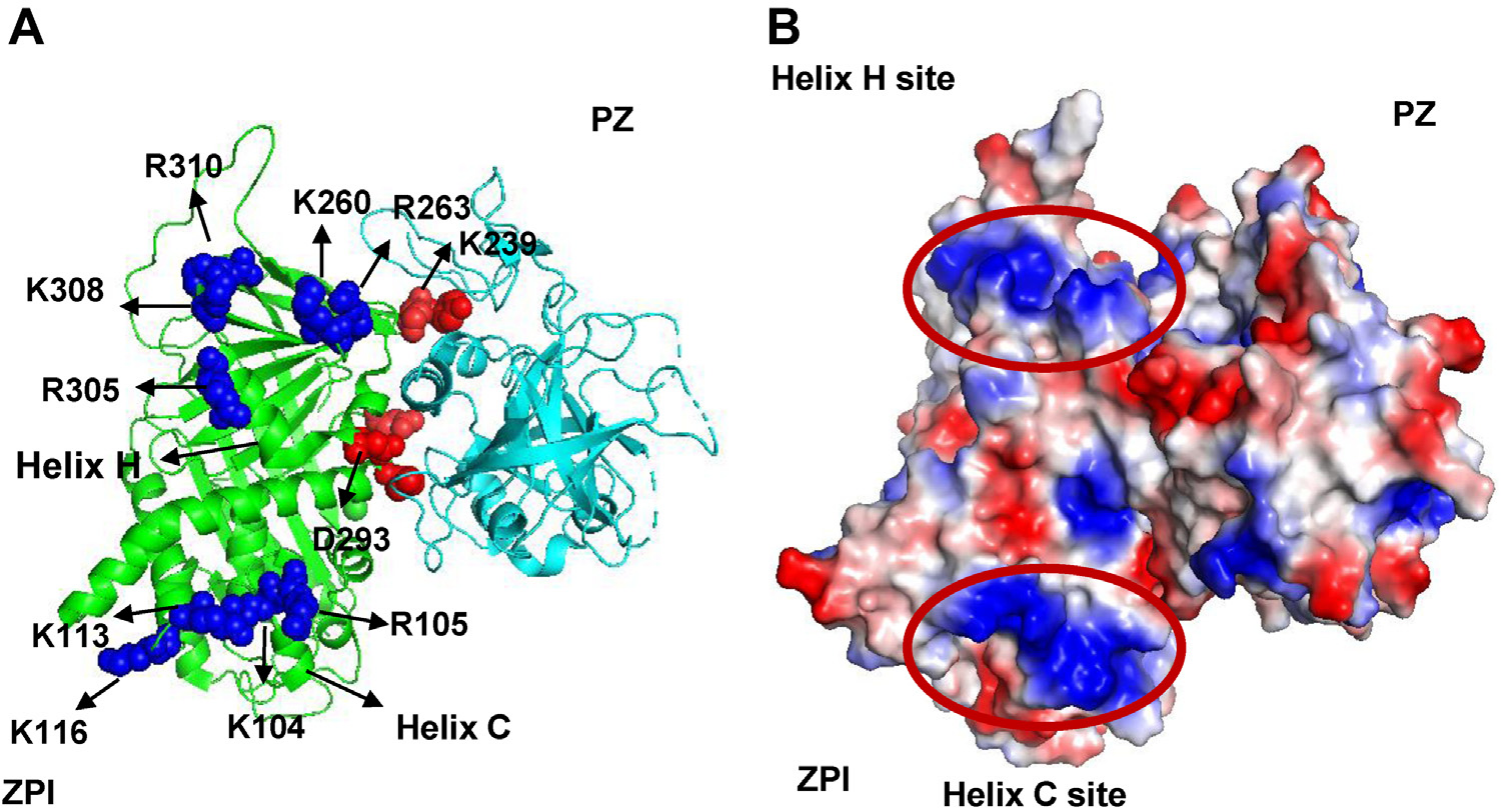
**Two discrete sites on ZPI bind heparin.***A*, ZPI basic residues shown to bind full-length heparin chains and to accelerate ZPI inhibition of FXa are depicted as *blue spheres* in a *ribbon* representation of the ZPI–PZ complex crystal structure (PDB: 3F1S) with ZPI in *green* and PZ in *cyan*. PZ binding residues are shown as *red spheres*. The two distinct sites implicated in heparin binding are the helix C site: R105, K104, K113, and K116 and the helix H site: R263, K260, R305, K308, and R310. *B*, surface charge representation of the ZPI–PZ complex structure in an identical orientation reveals the two positive patches comprising the helix C and helix H sites in *blue*, aligned on the same side of ZPI. PDB, Protein Data Bank; PZ, protein Z; ZPI, protein Z-dependent protease inhibitor.

Three of these residues (R305, R308, and R310) reside between the C terminus of helix H and the N terminus of strand 2 of sheet C, close to the helix H heparin-binding site of the related A clade serpin, PCI (5, 6). The other two residues (K260 and R263) reside in a loop connecting strand 3 of sheet C and strand 2 of sheet B that is structurally contiguous to helix H and that is partly conserved in the heparin-binding site of PCI (Figs. 5 and 7*A*). To confirm the importance of the helix H region of ZPI for heparin binding, we grafted the helix H-binding site of PCI onto the homologous helix H residues of ZPI (Fig. 5). Interestingly, this ZPI mutant showed an approximately twofold greater accelerating effect than WT (Table 4 and Fig. 6), confirming the helix H region as a functional heparin-binding site whose affinity for heparin could be augmented by mimicking the heparin-binding site of PCI.

### Helix C is also critical for the heparin cofactor function of ZPI

The previous study that mapped the heparin-binding site of ZPI to helix C/D employed mostly a chimeric approach in which the helix C and helix D secondary structures (including the connecting loop between helix C and D) were substituted with the homologous regions in the nonheparin-binding serpin, α_1_-protease inhibitor (11). To confirm the importance of this site for heparin cofactor function, we chose a less perturbing alanine scanning mutagenesis approach in which only basic residues in the implicated helix C/D site were mutated. The mutation of the six basic residues in the helix C/D site, K104A/R105A/K113A/K116A/K125A/R128A, resulted in a major reduction in the heparin acceleration from 75-fold to ∼7-fold (Table 4 and Fig. 5), confirming the importance of these residues for heparin cofactor function. The relative importance of helix C and D residues was further assessed by mutating helix D and helix C plus intervening loop residues alone. Notably, mutation of helix D residues, K125A/R128A, had no significant effect on heparin acceleration (∼89-fold) relative to WT, whereas mutation of the four helix C/intervening loop residues, K104A/R105A/K113A/K116A, produced the same major reduction in heparin acceleration (75-fold to ∼8.6-fold) as that of the combined helix C/D site mutations (Table 4). Helix D thus does not appear to contribute to the heparin-binding function of this site. Interestingly, this helix C site and the newly identified helix H site are aligned on the same face of ZPI, suggesting that the two sites may form an extended site for binding full-length heparin chains (Fig. 7).

### Relative contributions of helix C and helix H sites to heparin affinity for ZPI

**Figure 8:**
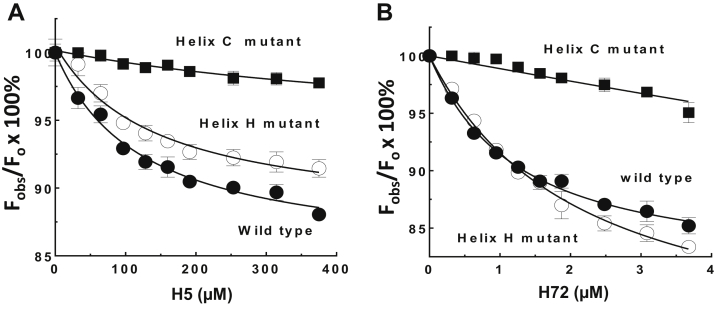
**Heparin binds stronger to helix C site than to helix H site.** Heparin-binding titrations of 200 nM WT (●), 1 μM helix C site mutant (K104A/R105A/K113A/K116A, ■), or 200 nM helix H site mutant (K260A/R263A/R305A/K308A/R310A, ○) ZPIs monitored by protein fluorescence quenching at 340 nm (excitation, 280 nm) are shown for H5 binding (*A*) or for H72 binding (*B*). The data represent the average of three to five independent measurements, presented as mean ± SD. Some SD lines lie within the symbol. *Solid lines* are computer fits of the data by the quadratic binding equation with the quenching end point for the helix C mutant fixed at the WT end point. Other details are provided in the “Experimental procedures” section. ZPI, protein Z-dependent protease inhibitor.

To evaluate the relative contributions of helix C and helix H sites to heparin-binding affinity, we analyzed the elution of the mutant ZPIs from heparin-Sepharose. Consistent with previous results, the helix C site mutations substantially reduced heparin affinity, whereas surprisingly, mutating the newly identified helix H site had a more modest effect in reducing heparin affinity (Table 5). To verify these differential heparin affinities for the two heparin-binding sites, we investigated the binding of heparin to ZPI mutants in which either the helix C site or the helix H site was mutated using tryptophan fluorescence quenching to monitor binding (Fig. 8, *A* and *B*). Significantly, the helix C site mutant ZPI (K104A/R105A/K113A/K116A) exhibited a marked reduction of tryptophan fluorescence quenching without evidence of saturation compared with WT in titrations of the mutant ZPI with both H5 and H72. The observed fluorescence quenching induced in the helix C mutant ZPI by both heparins was well fit by the binding equation assuming a WT quenching end point, which suggested a 10-fold to 20-fold weaker affinity of either heparin for the mutant ZPI compared with WT (*K*_*D*_s of 1900 ± 200 μM for H5 binding and 10 ± 2 μM for H72 binding to the helix C site mutant ZPI *versus* 123 ± 23 μM for H5 binding and 1.1 ± 0.1 μM for H72 binding to WT). By contrast, titrations of the helix H site mutant (K260A/R263A/R305A/K308A/R310A) with the two heparins showed fluorescence quenching and heparin affinities similar to WT for both heparins (*K*_*D*_s of 142 ± 42 μM for H5 binding and 1.8 ± 0.2 μM for H72 binding to the helix H site mutant). These findings suggest that the ZPI affinity for heparin largely derives from binding to the helix C site and are consistent with the results from elution of the mutants from heparin-Sepharose.

**Table 5:** Relative affinities of WT and mutant ZPIs for heparin-Sepharose

ZPI mutant	NaCl concentration (M) required to elute protein from heparin-Sepharose
WT	0.41
Helix C site mutants	
K125A/R128A	0.38
K104R/R105A/K113A/K16A	0.29
K104A/R105A/K113A/K116A/K125A/R128A	0.26
Helix H site mutant	
K260A/R263A/R305A/K308A/R310A	0.38

ZPIs (∼25 μg) in 1 ml equilibrating buffer of 20 mM Tris, 0.2 M NaCl, 2.5 mM CaCl_2_, pH 7.4, were applied to a 1 ml HiTrap heparin column and eluted with a salt gradient from 0.2 to 0.55 M NaCl with continuous monitoring of the eluant by protein fluorescence.

Salt concentrations corresponding to the peak elution position for the protein were determined from the elution volume as described in the “Experimental procedures” section. Based on replicate runs, errors in salt concentrations were estimated to be ±0.01 M.

## Discussion

While ZPI was initially characterized as a serpin whose anticoagulant function was dependent on PZ (ZPI naturally circulates as a tight complex with PZ in plasma (8)), it was later shown that heparin was an additional major physiologic activator of ZPI anticoagulant function (10). In this regard, ZPI resembles the anticoagulant serpins, AT, HCII, PN-1, and PCI, which all require heparin to inhibit their target coagulation proteases at physiologically significant rates. Like these serpins, heparin accelerates ZPI–protease reactions through a template bridging mechanism wherein heparin binding to both ZPI and the protease promotes the ternary complex encounter of the serpin with protease. Unlike these serpins, however, the heparin-binding site of ZPI was mapped to the helix C/D region of ZPI (11), a site distinct from the site utilized by other anticoagulant serpins. Thus, AT, HCII, and PN-1 bind heparin through a conserved site in helix D, whereas PCI binds heparin in the helix H region (1). Notably, the helix C/D site of ZPI is far removed from the PZ-binding site, which is centered on helix G, an observation that previously suggested that both heparin and PZ might bind ZPI simultaneously and form a ternary ZPI–PZ–heparin complex (11). It was therefore surprising to recently find that when we subjected the ZPI–PZ complex to size-exclusion chromatography in the absence and presence of heparin, we found that heparin induced the dissociation of the complex, suggesting that heparin binding to ZPI antagonized PZ binding (14).

In the present study, we have followed up this finding to convincingly demonstrate that heparin is indeed an antagonist of PZ binding to ZPI. While ZPI but not PZ alone was found to bind avidly to heparin-Sepharose, PZ was unable to bind to the resin even when complexed with ZPI. This suggested that binding of the ZPI–PZ complex to heparin-Sepharose through ZPI induces PZ to dissociate from the complex. Studies with an NBD-labeled ZPI, which report PZ binding by undergoing a ∼300% enhancement in NBD fluorescence, further showed a complete quenching of the enhanced fluorescence of the ZPI–PZ complex when the complex was titrated with heparin, consistent with heparin displacing PZ from the complex. Heparin likewise was shown to reduce the affinity of PZ for ZPI as measured by direct titrations of NBD-ZPI with PZ in the absence and presence of heparin. Significantly, heparins as small as five saccharides were effective at displacing PZ from the ZPI–PZ complex with the efficacy of heparins improving progressively with heparins of increasing chain length because of an increasing affinity of longer heparins for ZPI.

To understand how heparin antagonizes PZ binding, we considered the possibility that the previous mapping of the heparin-binding site had missed a heparin-binding region close to the PZ-binding site whose occupation by heparin directly interfered with PZ binding. Such an explanation was plausible given that mutation of the previous site did not completely abolish heparin binding and activation of ZPI (11). We thus employed the computer docking program, CLUSPRO, with demonstrated success in mapping the heparin-binding sites of numerous proteins known to bind heparin (16, 19) to search for an additional heparin-binding site. The docking revealed not only the previously mapped helix C region but also a new favored site near helix H close to the heparin-binding site of the related A clade serpin, PCI.

Mutagenesis of basic residues in the predicted new helix H site confirmed the importance of these residues in heparin acceleration of ZPI inhibition of FXa. The related A clade serpin, PCI, uses a somewhat shifted site in helix H to bind heparin and accelerate PCI reactions with its target proteases. Grafting the PCI helix H basic residues, which bind heparin onto the homologous residues in ZPI, was found to enhance the heparin-accelerating effect on the mutant ZPI–FXa reaction, consistent with the helix H region of ZPI being a functional heparin-binding site.

Since the previous mapping of the helix C/D site had mostly used a chimeric approach and replaced larger secondary structures of the C and D helices with the corresponding regions of the nonheparin-binding serpin α_1_-protease inhibitor, we reexamined the importance of the basic residues in this site. We confirmed the importance of the helix C and intervening loop residues but not the helix D residues for heparin acceleration of ZPI–protease reactions, consistent with the predictions of the docking program. Both sites thus seem important for heparin acceleration. Notably, the basic residues in the two sites form positive patches, which lie on the same face of ZPI (Fig. 7*B*), suggesting that both sites are involved in binding the long-chain heparins (>18 saccharides), which are required to accelerate ZPI–protease reactions (10). Unfortunately, we were unable to confirm whether complete abrogation of heparin acceleration could be achieved by mutating both sites since such a mutant was not expressable, possibly because of the cumulative effects of the destabilizing mutations K308A, R310A, and R128A (as analyzed by Site Directed Mutator, a server for predicting effects of mutations on protein stability (20)). It is also possible that other basic residues predicted to bind heparin in the helix C site whose contribution to heparin binding has not been tested (Fig. 4) may account for the inability to completely abrogate heparin acceleration.

Interestingly, heparin appeared to bind stronger to the helix C site than the helix H site based on the relative elution from heparin-Sepharose of ZPI variants with mutations in each site. This differential heparin affinity for the two sites was confirmed by intrinsic protein fluorescence quenching titrations of heparin binding to the helix C or helix H site mutants. Heparin affinity for ZPI thus derives largely from binding to the helix C site with a modest affinity augmentation in binding to the helix H site. Notably, the titrations revealed that the fluorescence quenching signaling heparin binding to ZPI could be induced by the binding of H5 or long-chain H72 either to the helix C site or to the helix H site. The binding of an H5 to ZPI through the higher affinity helix C site is thus sufficient to induce protein fluorescence quenching and to antagonize PZ binding (Fig. 2*B*) at the more distant PZ-binding site. This would imply that the protein fluorescence quenching and altered PZ binding accompanying heparin binding to ZPI results from allosterically induced global conformational changes in ZPI rather than proximity effects of heparin binding close to the PZ-binding site.

Intriguingly, only the helix H site is conserved in vertebrate ZPIs suggesting that for other vertebrate serpins, only the low-affinity helix H site exists, and thus, heparin may play a decreased role in activating ZPI in these species. This is supported by our finding that the docking program for locating the heparin-binding site in mouse ZPI suggested only the helix H site. The two-site model for binding long-chain heparins thus appears to be unique to human ZPI. We would also note that the prediction of both helix H and helix C heparin-binding sites of human ZPI utilized the ZPI chains from two ZPI–PZ complex structures since the structure of uncomplexed human ZPI has not been reported. PZ has been shown to induce limited conformational changes in ZPI, which appear to play a minimal role in promoting ZPI–PZ complex inhibition of FXa on a procoagulant membrane (13) but could function to antagonize heparin binding. Such reciprocal effects of PZ binding to ZPI on heparin binding could not be determined because of the inability to study heparin binding to ZPI in the presence of PZ using intrinsic protein fluorescence changes. An altered conformation of ZPI in the ZPI–PZ complex structure that reduces ZPI affinity for heparin could explain why the docking program predicted potential heparin-binding sites other than helix C and helix H, although such sites were less favored.

In summary, our study has shown that heparin acts as an allosteric activator of ZPI by binding to an extended site and inducing global conformational changes in the serpin, which both accelerate ZPI inhibition of FXa and antagonize the binding and activation of ZPI by PZ. Thus, only heparin or PZ cofactors can be functional at a given time and location under physiologically relevant conditions. ZPI circulates in plasma primarily as a complex with PZ with a small fraction free (8). This complex is poised to bind to an activated platelet or an endothelial cell membrane through the γ-carboxyglutamic acid domain of PZ at a site of vascular injury and thereby inhibit FXa generated on the membrane at the fastest rate. However, FXa, which escapes from procoagulant membrane sites, may also be inhibited by free ZPI when bound to vascular heparan sulfate or heparin glycosaminoglycans at the injury site at a physiologically significant rate. If the circulating ZPI–PZ complex binds to these localized glycosaminoglycans, it will release PZ and spare the cofactor for binding to circulating-free ZPI. Such a mechanism would retain PZ cofactor function to enable ZPI to most efficiently inhibit FXa on a procoagulant membrane but would also permit heparin-bound ZPI to rapidly inhibit FXa released from the membrane and thereby ensure localization of blood clotting at the injury site and limit clotting in a time-dependent and space-dependent manner.

## Experimental procedures

### Proteins

Recombinant ZPI and mutants were expressed in *Escherichia coli* and purified to homogeneity as previously reported (12). DNA mutagenesis was carried out by PCR using the Quick-Change Mutagenesis kit (Stratagene). All mutations were confirmed by DNA sequencing. A single cysteine ZPI (K239C) with two native cysteines (C264 and C169) mutated to serine and alanine, respectively, was labeled with NBD fluorophore as previously described (15). Human plasma PZ and FXa were purchased from Enzyme Research Laboratories. All proteins were judged >95% pure by SDS-PAGE analysis. Molar concentrations of recombinant ZPI and plasma PZ were determined from the absorbance at 280 nm using absorption coefficients calculated from the amino acid sequence (15). FXa concentrations were determined by a standard assay with chromogenic substrate that was calibrated with protease of known active-site concentration (18).

### Phospholipids

Small and unilamellar phospholipid vesicles were prepared from a 7:3 mixture (by weight) of dioleyl phosphatidylcholine and dioleoyl phosphatidylserine (Avanti Polar Lipids) as described previously (18).

### Heparin

Heparins with a narrow molecular weight distribution and average chain length of 26 to 72 saccharides were purified from porcine mucosal heparin (Diosynth) by repeated gel exclusion chromatography and affinity fractionation for AT, as described previously (21). Oligosaccharide heparins from 4 to 18 saccharides in length were a generous gift of Ingemar Björk and prepared by chemical degradation of commercial heparin followed by chromatographic purification (22). An exception was a synthetic H5 (fondaparinux), which was a gift of Sanofi. Molar concentrations of heparins were determined from the weight concentration obtained by an Azure A dye–binding assay together with the molecular weight (21).

### ZPI–PZ binding to heparin-Sepharose analyzed by SDS-PAGE and immunoblotting

Samples of 500 nM ZPI, ZPI–PZ complex, or PZ in 200 μl of 50 mM Tris buffer (pH 7.4), 0.1 M NaCl, and 2.5 mM CaCl_2_ were mixed with 50 μl heparin-Sepharose resin equilibrated with the same buffer and then transferred to a small column with the outlet closed. After incubation for 40 min at room temperature, the outlet was opened and the unbound proteins were allowed to flow through the column. The flowthrough was collected, and the columns were then washed three times with 20 volumes of equilibrating buffer. The wash buffer was discarded, and the resins were resuspended in 200 μl of the same buffer. The flow-through and resuspended resin were mixed with SDS sample buffer containing 100 mM dithiothreitol, heated at 100 °C for 5 min, and then subjected to 10% SDS-PAGE. The proteins were transferred to a polyvinylidene difluoride membrane and analyzed by anti-ZPI (Sigma–Aldrich) and anti-PZ (Haematologic Technologies) antibodies.

### Fluorescence emission spectra and equilibrium binding of PZ or heparin to labeled and unlabeled ZPI

Fluorescence emission spectra of NBD-ZPI (50–100 nM) were measured in the absence or the presence of PZ and heparin in 20 mM Tris buffer (pH 7.4), 0.2 M NaCl, 0.1% PEG 8000, 1 mg/ml bovine serum albumin (BSA), and 2.5 mM CaCl_2_ with an SLM 8000 spectrofluorometer with excitation at 480 nm over the emission range of 500 to 600 nm. Intrinsic protein fluorescence of WT and mutant ZPIs (200 nM to 1 μM) was measured in the same buffer without BSA, with excitation at 280 nM over the emission range of 300 to 400 nm. Emission was monitored in 5-nm steps with 5 to 10 s of integrations per step. Spectra were obtained in triplicate and averaged. Buffer, buffer plus PZ, or buffer plus heparin background spectra were subtracted, and dilution corrections were made for added PZ or heparin (<10%).

Equilibrium binding of heparin to NBD-ZPI was measured by competitive binding titrations in which heparins of various lengths were titrated into NBD-ZPI (50 nM) complexed with a molar excess of PZ (62 nM) in a lower ionic strength buffer (0.1 M NaCl) and heparin binding followed by the quenching of NBD-ZPI–PZ complex fluorescence. Titrations were computer fit by the competitive binding equation,

$$\frac{\Delta F_{\text{max}}\;-\;\Delta F_{\text{obs}}}{\Delta F_{\text{max}}}=\frac{F_{\text{obs}}\;-\;F_{\infty }}{F_{0}\;-\;F_{\infty }}=\frac{{\left[\text{ZPI}\right]}_{0}\;+\;{\left[\text{PZ}\right]}_{0}\;+\;K_{D,\text{PZ}}\;\times \;\left(1\;+\;\frac{{\left[\text{H}\right]}_{0}}{K_{D,H}}\right)\;-\;\sqrt{{\left({\left[\text{ZPI}\right]}_{0}\;+\;{\left[\text{PZ}\right]}_{0}\;+\;K_{D,\text{PZ}}\;\times \;\left(1\;+\;\frac{{\left[\text{H}\right]}_{0}}{K_{D,H}}\right)\right)}^{2}\;-\;4\;\times \;{\left[\text{ZPI}\right]}_{0}\;\times \;{\left[\text{PZ}\right]}_{0}}}{{\left[\text{ZPI}\right]}_{0}\;+\;{\left[\text{PZ}\right]}_{0}\;+\;K_{D,\text{PZ}}\;-\;\sqrt{{\left({\left[\text{ZPI}\right]}_{0}\;+\;{\left[\text{PZ}\right]}_{0}\;+\;K_{D,\text{PZ}}\right)}^{2}\;-\;4\;\times \;{\left[\text{ZPI}\right]}_{0}\;\times \;{\left[\text{PZ}\right]}_{0}}}$$

in which Δ*F*_obs_ is the observed change in fluorescence of PZ complexed and uncomplexed NBD-ZPI before (*F*_o_) and after heparin addition (*F*_obs_), Δ*F*_max_ is the maximal fluorescence change upon reaching the end point fluorescence of NBD-ZPI after complete dissociation of PZ (*F*_∞_), [ZPI]_o_, [PZ]_o_, and [H]_o_ are total concentrations of NBD-ZPI, PZ, and heparin, respectively, and *K*_*D*,PZ_ and *K*_*D*,H_ are dissociation constants for the NBD-ZPI–PZ and the NBD-ZPI–heparin interactions, respectively. This quadratic equation is derived from the cubic competitive binding equation (23) based on the condition that heparin concentrations were always in large molar excess over ZPI so that the concentration of heparin bound to ZPI was small relative to the total heparin concentration. In fitting data to the aforementioned equation, *K*_*D*,PZ_ was fixed at the value determined by titration of NBD-ZPI with PZ in the absence of heparin, and *K*_*D*,H_ was the fitted parameter.

The effect of heparin on PZ binding to ZPI was measured by titrations of 50 nM NBD-ZPI with PZ in the absence and presence of different fixed levels of heparins of various lengths in the higher ionic strength buffer used for emission spectra. Titrations were monitored from the enhancement of NBD-ZPI fluorescence that signals PZ binding. These titrations were fit by the quadratic binding equation,

$$\frac{\Delta F_{\text{obs}}}{F_{0}}=\frac{\Delta F_{\text{max}}}{F_{0}}\times \frac{{\left[\text{ZPI}\right]}_{0}\;+\;{\left[\text{PZ}\right]}_{0}\;+\;K_{D,\text{PZapp}}\;-\;\sqrt{{\left({\left[\text{ZPI}\right]}_{0}\;+\;{\left[\text{PZ}\right]}_{0}\;+\;K_{D,\text{PZapp}}\right)}^{2}\;-\;4\;\times \;{\left[\text{ZPI}\right]}_{0}\;\times \;{\left[\text{PZ}\right]}_{0}}}{2\;\times \;{\left[\text{ZPI}\right]}_{0}}$$

where Δ*F*_obs_/*F*_o_ and Δ*F*_max_/*F*_o_ are the observed and maximal fluorescence changes after addition of PZ expressed relative to the initial free NBD-ZPI fluorescence, respectively, and *K*_*D*,PZapp_ = *K*_*D*,PZ_ × (1 + [H]_o_/*K*_*D*,H_) is the apparent dissociation constant for the ZPI–PZ interaction in the absence or presence of heparin. The fitted parameters were *K*_*D*,PZapp_ and Δ*F*_max_/*F*_o_.

Direct equilibrium binding of heparin to ZPI was measured by titrations of unlabeled WT and variant ZPIs (0.2–1 μM) with heparin monitored by the quenching of intrinsic protein fluorescence accompanying heparin binding at excitation and emission wavelengths of 280 and 340 nm, respectively. Titrations were computer fit by the aforementioned quadratic binding equation with [H]_o_ replacing [PZ]_o_ and *K*_*D*,H_ replacing *K*_*D*,PZapp_.

### ZPI–heparin docking analysis

The program CLUSPRO was used to virtually dock a heparin tetrasaccharide to the ZPI chain of the two available ZPI–PZ complex structures (PDB code: 3F1S and 3H5C) and to uncomplexed mouse ZPI (PDB code: 4AJT). The program evaluates a billion docking poses of a generic heparin tetrasaccharide with ZPI and clusters the 1800 lowest energy-docking positions based on ligand positions lying within a 9 Å RMSD radius in each cluster (16).

### Kinetics of ZPI–FXa reactions

Heparin accelerating effects on the kinetics of ZPI–FXa reactions were evaluated as a function of heparin concentration under pseudo–first-order conditions (*i.e.*, with a large molar excess of inhibitor over protease) by incubating reaction mixtures of 100 μl containing 70 nM ZPI and ∼0.5 nM FXa in the absence or presence of varying levels of H50 ranging from 10^−9^ to 10^−4^ M in 20 mM Tris buffer (pH 7.4), 0.1 M NaCl, 0.1% PEG 8000, 0.1 mg/ml BSA, and 2.5 mM CaCl_2_ at 25 ^°^C for reaction times of 2 to 5 min. Reactions were quenched by adding 1 ml of 50 μM Pefafluor FXa substrate (Centerchem, Inc) containing 50 μg/ml polybrene and 5 mM EDTA. The initial linear rate of substrate hydrolysis was monitored at an excitation wavelength of 380 nm and an emission wavelength of 440 nm. The observed pseudo–first-order rate constant for reactions (*k*_obs_) was calculated from the equation,

$$k_{\text{obs}}=\text{In}([v_{\text{o}}-v_{\infty }]/[v_{t}-v_{\infty }])/t$$

where v_*t*_ and *v*_∞_ are the initial velocities of protease hydrolysis of the substrate after reaction with ZPI for the fixed reaction time and after reaching the reaction end point, respectively, *v*_o_ is the velocity measured in control reactions without ZPI, and *t* is the fixed reaction time. The dependence of *k*_obs_ on heparin concentration was computer fit by nonlinear least squares analysis by the ternary complex bridging model equation (10).

Apparent second-order rate constants for unaccelerated and H50-accelerated ZPI–FXa reactions were measured in the absence and presence of the optimal H50 concentration under pseudo–first-order conditions by analyzing full ZPI–FXa reaction progress curves over the ZPI concentration range of 10 to 900 nM with ∼0.5 nM FXa. Full reaction progress curves were also measured for ZPI–FXa reactions in the presence of PZ equimolar with ZPI, 25 μM lipid, and 5 mM CaCl_2_, as a function of the ZPI concentration in the range of 10 to 40 nM. Identical reaction mixtures were quenched at varying reaction times with substrate, and the time course of protease inactivation was measured from the decrease in initial rates of substrate hydrolysis. The first-order rate of decrease in protease activity with time was computer fit by a single exponential decay function with a nonzero end point and *k*_obs_ obtained from the fitted exponential decay constant. Apparent second-order association rate constants (*k*_a,app_) were obtained from the slopes of linear plots of *k*_obs_ *versus* the ZPI concentration according to the equation,

$$k_{\text{obs}}=k_{d}+k_{\text{a,app }}\times \;{\left[\text{ZPI}\right]}_{\text{0}}$$

In which *k*_*d*_ represents the rate constant for ZPI–FXa complex dissociation and [ZPI]_o_ is the total ZPI concentration. *k*_*d*_ was fixed at the experimentally determined value of 10^−4^ s^−1^ for WT and ZPI mutants as described (18).

### Stoichiometries of ZPI reaction with FXa

Fixed concentrations of FXa (50–100 nM FXa) were reacted with increasing concentrations of ZPI, ranging from a approximately threefold to sevenfold molar excess, in the absence of cofactors or in the presence of either 1 μM H50 or PZ equimolar with ZPI, 25 μM lipid, and 5 mM CaCl_2_, in kinetics buffer at 25 ^°^C in reaction volumes of 50 μl. After allowing sufficient time to reach a reaction end point, based on measured second-order associate rate constants (∼5–30 min), remaining proteolytic activity was measured by reacting an aliquot (5–20 μl) of the reaction mixtures with 1 ml of 100 μM Spectrozyme fXa (American Diagnostica) containing 50 μg/ml Polybrene and 5 mM EDTA and monitoring the rate of absorbance change at 405 nm. The apparent SI was determined from the fitted abscissa intercept of a linear plot of residual protease activity against the molar ratio of inhibitor to protease (18). Because the ZPI–PZ complex dissociates to a small extent over the reaction time, apparent SIs depend on the time of incubation. To correct for such complex dissociation, apparent SIs were multiplied by the factor, exp (−*k*_*d*_ × *t*), where *k*_*d*_ is the measured complex dissociation rate constant of 10^−4^ s^−1^ and *t* the fixed reaction time. Simulations verified the validity of this correction for small extents of dissociation. Reported SIs are average values of at least two independent measurements at different reaction times.

### Relative affinities of ZPI variants for heparin-Sepharose

WT and mutant ZPIs (∼25 μg) in 1 ml equilibrating buffer of 20 mM Tris (pH 7.4), 0.2 M NaCl, and 2.5 mM CaCl_2_ were applied to a 1 ml HiTrap heparin column and eluted with a salt gradient from 0.2 to 0.55 M NaCl with continuous monitoring of the eluant by protein fluorescence. Salt concentrations corresponding to the peak elution position of the protein were calculated from the elution volume based on calibration of the volumes at the beginning and end of the gradient (10).

## Data availability

All data are contained within the article.

## Conflict of interest

The authors declare that they have no conflicts of interest with the contents of this article.
